# P-631. RSV Prophylaxis with *Nirsevimab* in Infants: Systematic Review of Early Real-World Evidence on Effectiveness and Impact

**DOI:** 10.1093/ofid/ofae631.828

**Published:** 2025-01-29

**Authors:** Moritz Wick, Anna C Meyer, Oliver Damm, Annika Wülfing, Oliver Martyn, Rolf Kramer, M Knuf

**Affiliations:** Sanofi-Aventis GmbH, Berlin, Berlin, Germany; Sanofi-Aventis GmbH, Berlin, Berlin, Germany; Sanofi-Aventis GmbH, Berlin, Berlin, Germany; Sanofi-Aventis GmbH, Berlin, Berlin, Germany; Sanofi Vaccines, Copenhagen, Hovedstaden, Denmark; Sanofi Vaccines, Lyon, France, Lyon, Rhone-Alpes, France; Klinikum Worms, Worms, Rheinland-Pfalz, Germany

## Abstract

**Background:**

Respiratory syncytial virus (RSV) is a leading cause of respiratory tract infections and hospitalizations among infants worldwide. In 2023, the universal RSV immunization of all infants with the novel monoclonal antibody nirsevimab was introduced in several countries. This systematic review aims to summarize early real-world evidence on achieved immunization rates as well as on the impact and effectiveness of nirsevimab prophylaxis among infants.

**Methods:**

A systematic literature search for full-text publications was conducted in the databases PubMed and Embase. The review included observational studies that investigated the impact or effectiveness of nirsevimab in routine use. Clinical research, decision analytic modeling studies, and studies describing the disease burden of RSV without direct reference to nirsevimab use were excluded.

**Results:**

Five studies fulfilled the inclusion criteria, including one from the United States (US), one from Luxembourg, and three studies from different regions in Spain. Population-wide immunization rates were reported in Luxembourg and several Spanish regions, all exceeding 80%. Four studies estimated the effectiveness of nirsevimab in preventing RSV-related hospitalizations, including two population-based cohort studies and two case-control studies. The effectiveness of nirsevimab in preventing RSV-related hospitalizations was calculated at 88.7% (95%-CI: 70-96), 84.4% (95%-CI: 77-90), and 82.0% (95%-CI: 66-90) in the Spanish studies and 90% (95 %-CI 75-96) in the US. While no effectiveness estimate was reported in Luxembourg, the number of RSV-related hospitalizations among infants under 6 months decreased by 69% after nirsevimab introduction compared to the same period of the previous year.

**Conclusion:**

High uptake was observed in both Luxembourg and Spain. Despite methodological differences between studies and varying implementation strategies between countries, observed effects of recommending nirsevimab to all infants were largely consistent. International evidence suggests that nirsevimab substantially reduces RSV-related hospitalizations and thus relieves the burden on healthcare systems during the RSV season.

**Disclosures:**

**Moritz Wick, MSc**, Sanofi: Employee|Sanofi: Stocks/Bonds (Public Company) **Anna C. Meyer, PhD**, Sanofi: Employee **Oliver Damm, PhD**, Sanofi: Employee **Annika Wülfing, PhD**, Sanofi: Employee and may hold stocks **Oliver Martyn, MPH**, Sanofi: Employee of Sanofi|Sanofi: Stocks/Bonds (Public Company) **Rolf Kramer, PhD**, Sanofi: Employee of Sanofi|Sanofi: Stocks/Bonds (Public Company)

